# HLA-G 3142C/G polymorphism is related to development of symptoms in HTLV-1 infected individuals

**DOI:** 10.1186/1742-4690-11-S1-P74

**Published:** 2014-01-07

**Authors:** Daiani C Cilião-Alves, Maurício C Rocha-Junior, Rodrigo Haddad, Virgínia MD Wagatsuma, Osvaldo M Takayanagui, Dimas T Covas, Simone Kashima, Eduardo A Donadi

**Affiliations:** 1 Faculdade de Medicina de Ribeirão Preto – USP, Ribeirão Preto, São Paulo, Brazil; 2 Fundação Hemocentro de Ribeirão Preto, Ribeirão Preto, São Paulo, Brazil; 3 Faculdade de Ciências Farmacêuticas de Ribeirão Preto – USP, Ribeirão Preto, São Paulo, Brazil; 4 Universidade de Brasília, Faculdade de Ceilândia – UNB/FCE, Brasília, Distrito Federal, Brazil

## 

The majority of HTLV-1 infected individuals remain asymptomatic (HAC) throughout life, and the risk factors associated to the development of related diseases, such as HAM/TSP and ATL, are not fully understood. The human leukocyte antigen-G (HLA-G) is expressed in several pathological conditions and it has been associated to immunosuppressive effects allowing the virus-infected cells to escape of the host antiviral defense. Here, we evaluated the correlation between HLA-G polymorphisms in symptomatic and asymptomatic HTLV-1 infected individuals. Four polymorphisms of the exon 8 of the HLA-G gene were analysed by gene sequencing in HAC (n=48) and HAM/TSP patients (n=43). No difference was found between the groups for 3035C/T, 3187A/G and 3196C/G polymorphisms. On the other hand, despite of the absence of statistical significance, HAC group showed higher frequency of the 3142GG genotype (responsible for decreased transcription of theHLA-G) compared to HAM/TSP group (p=0,0607). Still, HAC group showed lower frequency of the 3142CC genotype (responsible for increased transcription of the HLA-G) compared to HAM/TSP group (p=0,0762). Furthermore, HAC group showed statistically higher frequency of the 3142G allele and lower frequency of the 3142C allele compared to HAM/TSP group (p=0,0244). In conclusion, HTLV-1-related symptoms in HAM/TSP group could be partially determined by higher expression of HLA-G (caused by higher frequency of the 3142C allele). We hypothesise that the higher expression of HLA-G protects the HTLV-1 infected cell against immune system attack the increasing proviral load and trigger the HAM/TSP symptoms.

## Financial support

FUNDHERP, FAPESP and CNPq.

